# INHBA is a prognostic predictor for patients with colon adenocarcinoma

**DOI:** 10.1186/s12885-020-06743-2

**Published:** 2020-04-15

**Authors:** Xueying Li, Weiming Yu, Chao Liang, Yuan Xu, Miaozun Zhang, Xiaoyun Ding, Xianlei Cai

**Affiliations:** 1 Department of Gastroenterology, Ningbo First Hospital, Ningbo, China; 2 Department of Gastrointestinal Surgery, Ningbo Medical Center Lihuili Hospital, Ningbo, China

**Keywords:** Colon adenocarcinoma, Bioinformatics analysis, INHBA, Nomogram

## Abstract

**Background:**

Colon adenocarcinoma (COAD) is one of the most lethal cancers. It is particularly important to accurately predict prognosis and to provide individualized treatment. Several lines of evidence suggest that genetic factors and clinicopathological characteristics are related to cancer onset and progression. The aim of this study was to identify potential prognostic genes and to develop a nomogram to predict survival and recurrence of COAD.

**Methods:**

To identify potential prognostic genes in COAD, microarray datasets were downloaded from the Gene Expression Omnibus (GEO) database. Differentially expressed genes (DEGs) were obtained from GEO2R. Venn diagram was drawn to select those genes that were overexpressed in all datasets, and survival analyses were performed to determine the prognostic values of the selected genes. New nomograms were developed based on the genes that were significantly associated with prognosis. Clinicopathological data were obtained from The Cancer Genome Atlas (TCGA). Finally, the new nomograms were compared head-to-head comparison with the TNM nomogram.

**Results:**

From GSE21510, GSE110223, GSE113513 and GSE110224, a total of 834, 218, 236 and 613 overexpressed DEGs were screened out, respectively. The Venn diagram revealed that 12 genes appeared in all four profiles. After survival analyses, only INHBA expression was associated with both overall survival (OS) and disease-free survival (DFS). Multivariate analyses revealed that age, pathological N and pathological M were significant independent risk factors for OS. Age, pathological N, pathological M and INHBA were significant independent risk factors for DFS. Two prediction models predicted the probability of 3-year survival and 5-year survival for OS and DFS, respectively. The concordance indexes were 0.785 for 3-year overall survival, 0.759 for 5-year overall survival, 0.789 for 3-year disease-free survival and 0.757 for 5-year disease-free survival. The head-to-head comparison according to time-dependent ROC curves indicated that the new models had higher predictive accuracy. Decision curve analyses (DCA) indicated that the clinical value of the new models were higher than TNM models for predicting disease-free survival.

**Conclusion:**

The combination of INHBA expression with a clinical nomogram improves prognostic power in colon adenocarcinoma, especially for predicting recurrence.

## Background

Colorectal cancer (CRC) ranks second among the world’s top ten cancers [1]. In 2018, more than 1.8 million new CRC cases were diagnosed and 880,000 cancer-related deaths occurred worldwide [2]. In China, the incidence and mortality of CRC rank fifth and fourth, respectively [3]. Colon adenocarcinoma is one of the most common types of CRC [4] and has become more prevalent in recent years [5]. Comprehensive treatment based on multidisciplinary discussion has become the trend of CRC treatment. Despite the fact that surgery combined with chemotherapy, as well as targeted therapy and immunotherapy have improved prognosis, the overall efficacy, especially long-term and high-quality survival remain unsatisfactory.

The prognosis of colon adenocarcinoma primarily depends on the extent of disease. Nevertheless, various prognostic factors have been observed in addition to American Joint Committee on Cancer (AJCC) Tumor Node Metastasis (TNM) stage. There prognostic factors include age, gender and gene expression [6, 7]. With the rapid development of gene sequencing technology, GEO and TCGA have been playing increasing important roles in bioinformatics analysis [8, 9]. These databases provide sequencing data for discovery of new functional genes and for analyzing the effect of these genes on prognosis. These analyses require a new method, combining clinical characteristics and gene information; a nomogram is a good tool for this purpose.

Therefore, the aim of this study was to identify prognostic genes using comprehensive bioinformatic analysis and to develop a nomogram to predict the overall survival and disease-free survival of patients with COAD based on GEO and TCGA databases.

## Methods

### Microarray data

In the discovery step, we identified datasets that compared mRNA expression in CRC tissue with that of normal tissue. Gene expression profiles of GSE21510 (with 148 samples), GSE110223 (with 26 samples), GSE113513 (with 28 samples) and GSE110224 (with 34 samples) were obtained from the National Center for Biotechnology Information (NCBI) GEO database (https://www.ncbi.nlm.nih.gov/geo/). GSE21510 and GSE11024 datasets were based on the GPL570 platform, while GSE110223 was based on the GPL96 platform and GSE 113513 was based on the GPL15207 platform.

### Screening for integrated differentially expressed genes (DEGs)

GEO2R, the tool provided by the GEO database that depends on R package ‘limma’ was used to identify DEGs in each dataset. Adjusted *p* values < 0.05 and log_2_FC > 1 were set as cut-off criteria for screening out the overexpressed DEGs. The list of significantly up-regulated genes was exported separately.

A Venn diagram containing four lists of up-regulated genes was drawn online (http://bioinformatics.psb.ugent.be/webtools/Venn/) to identify those genes that were overexpressed in all datasets. All expression levels of selected genes were verified in TCGA (http://ualcan.path.uab.edu/) [10]. We drew a heatmap describing levels of potential hub gene expression.

### Kaplan - Meier survival analysis

The prognostic values of selected genes were analyzed. Gene expression profiling and interactive analyses (GEPIA) [11] were used for survival analyses (http://gepia.cancer-pku.cn/). The GEPIA contains 9736 tumors and 8587 normal samples from TCGA and GTEx. Kaplan - Meier plots of OS and DFS were drawn and hazard ratios (HRs) were calculated for each selected gene individually. Log rank *p*-values were presented, and those genes significantly associated with prognosis were entered into the next stage of model building.

### Clinical and bioinformatic information

TCGA was accessed on May 5, 2019, and a total of 459 COAD patient clinical data with tumors’ RNA expression data were collected (https://tcga-data.nci.nih.gov/). Clinical parameters included gender, age, pathologic T stage, pathologic N stage, pathologic M stage, vital status, recurrent status and follow-up period (days). Considering the influence of surgical factors, we excluded those cases whose follow-up time were less than 30 days. Median RNA expression value was regarded as the cut-off to divide patients into high or low expression groups.

### Development of risk prediction model

According to TCGA data, we developed a nomogram combing gene expression with clinical information (new model) for prediction of survival and recurrence at 3 years and 5 years in individual COAD patients. Another nomogram based on pathologic TNM stage (TNM model) was developed for head-to-head comparison with the first comprehensive model.

### Statistical analysis

We used the Cox proportional hazard regression model to estimate hazard ratio (and its 95% confidence interval (CI)) for each potential risk factor. Inclusion and exclusion criteria of type I error = 0.1 were set in the stepwise multivariate Cox regression analysis.

The discrimination reflects the ability of a model to distinguish events and non-events correctly, and these were validated using C-statistics. The Concordance index (C-index) is analogous to the area under the receiver operating characteristic (ROC) curve. The predictive capacity of models was summarized using time-dependent ROC curves [12, 13]. The calibration refers to the closeness between the predicted probabilities and the actual outcomes, and this was validated using calibration plots [14]. To test the clinical value of the predictive new model, we generated a decision curve analysis graph to visualize the potential net benefit between two models at each threshold probability [15, 16].

To calculate sample size, we set expected sensitivity as 70%, expected specificity as 90%, permissible error of sensitivity and specificity both as 5%, and alpha (two-sided) as 0.05 [17, 18]. After calculation, the expected sample size was 323. This demonstrated that number of TCGA patients was sufficient for this study.

A two-sided *p*-value of <0.05 was considered statistically significant. All statistical analyses were conducted using R software for Windows, version 3.6.1.

## Results

### Identification of DEGs in COAD

**Fig. 1:**
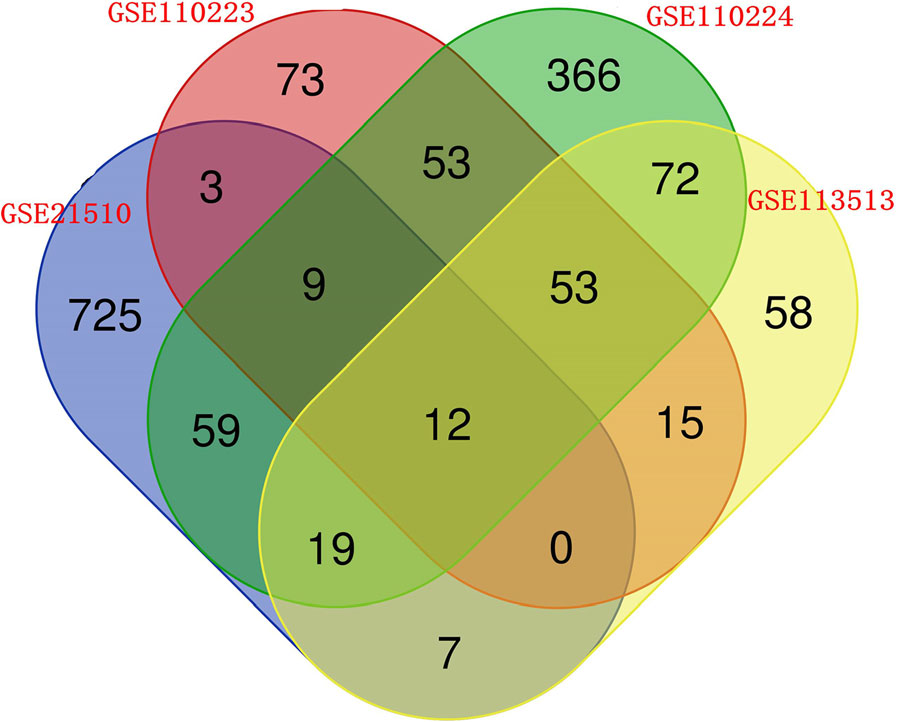
Venn diagram containing four lists of up-regulated genes

**Fig. 2:**
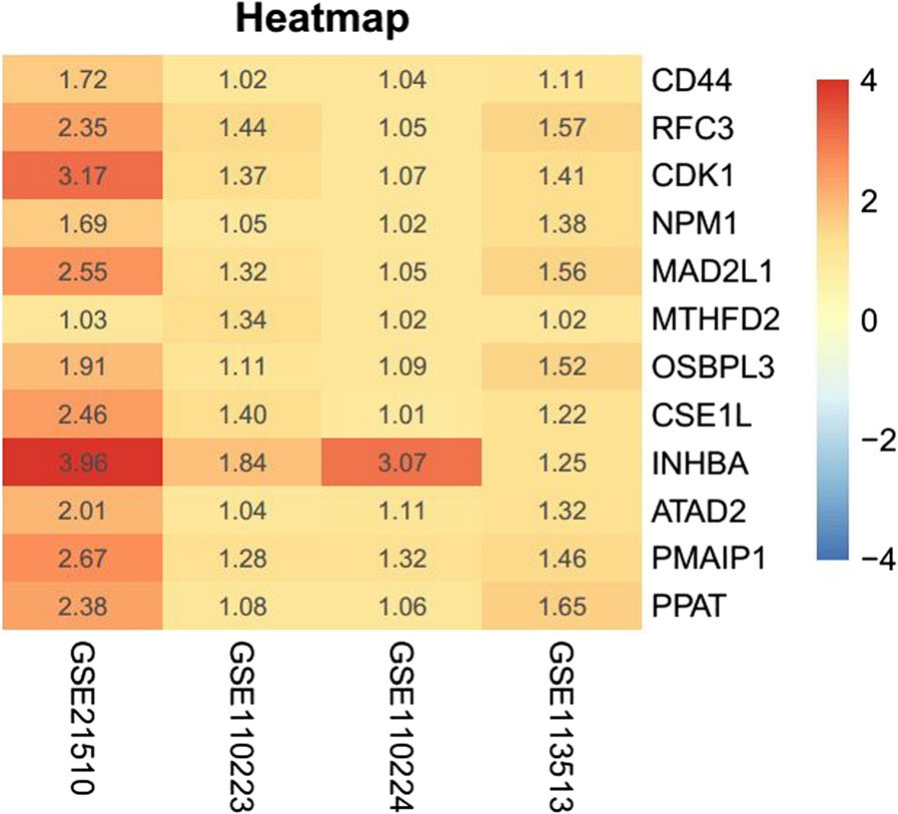
Heatmap describing the level of potential hub genes expression

We downloaded four COAD gene expression profiles (GSE21510, GSE110223, GSE113513 and GSE110224) from the GEO database and screened out 834, 218, 236 and 613 overexpressed DEGs respectively using GEO2R. A Venn diagram was generated (Fig. 1) and 12 genes (CD44, RFC3, CDK1, NPM1, MAD2L1, MTHFD2, OSBPL3, CSE1L, INHBA, ATAD2, PMAIP1 and PPAT) that were overexpressed in the four profiles were discovered. The 12 selected genes were verified in TCGA and charted on a heat map (Fig. 2).

### Survival analysis

**Fig. 3:**
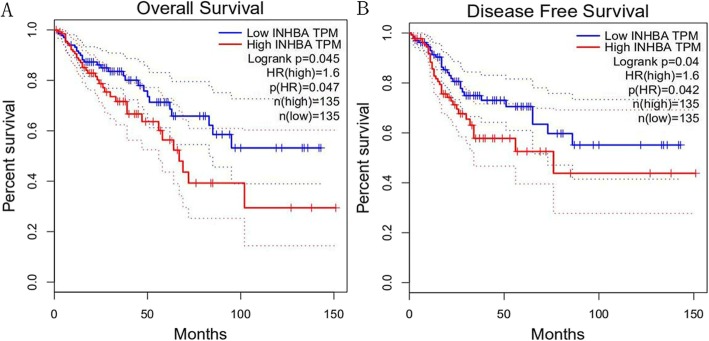
Kaplan - Meier plots of INHBA expression: **a**. overall survival; **b**. disease free survival

To further explore the survival values of the 12 selected genes, we drew Kaplan - Meier curves of OS and DFS according to gene expression. Only INHBA exhibited statistical significance in both OS and DFS curves (Fig. 3, log-rank *p* = 0.045 and 0.040, respectively). High levels of INHBA expression were associated with poor prognosis, while the other eleven genes did not present prognostic prediction values for OS or DFS (Supplementary Figure 1 and Supplementary Figure 2). Therefore, only INHBA was entered into the subsequent stage of model building.

### Baseline patient characteristics in TCGA

Clinicopathological data were integrated with INHBA expression levels. Median of INHBA expression was used to divide patients into high-INHBA groups and low- INHBA groups. Patients who had a follow-up time less than 30 days or had no clinical overall survival or disease-free survival information were excluded. Finally, data on 420 COAD patients for OS and data on 388 COAD patients for DFS were obtained. Detailed baseline characteristics are summarized in Table 1.

**Table 1:** Clinicopathological characteristics of the development set

Factors	Subgroup	OS set (*n* = 420) No of patient (%)	DFS set (*n* = 388) No of patient (%)
Age	<65	165 (39.3)	156 (40.2)
	65–74	127 (30.2)	118 (30.4)
	≥75	128 (30.5)	114 (29.4)
Gender	Male	226 (53.8)	212 (54.6)
	Female	194 (46.2)	176 (45.4)
pT	T1	11 (2.6)	11 (2.8)
	T2	74 (17.6)	72 (18.6)
	T3	286 (68.1)	268 (69.1)
	T4	49 (11.7)	37 (9.5)
pN	N0	249 (59.3)	239 (61.60)
	N1	99 (23.6)	91 (23.5)
	N2	72 (17.1)	58 (14.9)
	N3	0 (0)	0 (0)
pM	M0	364 (86.7)	347 (89.4)
	M1	56 (13.3)	41 (10.6)
INHBA	Low	255 (60.7)	239 (61.6)
	High	165 (39.3)	149 (38.4)

### Risk factors for overall survival and disease-free survival

In the univariate Cox analysis, age, pathological T, pathological N, pathological M and INHBA were all associated with overall survival and disease-free survival (Table 2). By contrast, gender did not have a significant effect. The significant risk factors determined in the univariate analysis were used in multivariate Cox analysis. Finally, age, pathological N and pathological M emerged as significant independent risk factors for OS, while age, pathological N, pathological M and INHBA were significant independent factors for DFS (Table 2).

**Table 2:** Risk factors for overall survival and disease free survival according to Cox proportional hazards regression model

Factors	Subgroup	OS set(*n* = 420)					DFS set(*n* = 388)						
		Univariate analysis			Multivariate analysis		Univariate analysis				Multivariate analysis		
		HR	95%CI	p	HR	95%CI	p	HR	95%CI	p	HR	95%CI	p
Age	<65	1			1			1			1		
	65–74	0.99	0.55–1.75	0.960	1.29	0.72–2.32	0.393	0.47	0.27–0.81	**0.007**	0.50	0.28–0.88	**0.016**
	≥75	1.86	1.13–3.09	**0.016**	2.28	1.36–3.83	**0.002**	0.90	0.60–1.57	0.897	1.23	0.75–2.02	0.421
Gender	Male	1						1					
	Female	1.18	0.55–1.31	0.466				0.75	0.49–1.16	0.199			
pT	T1	1			1			1			1		
	T2	1.62	0.06–5.96	0.676	0.46	0.05–4.48	0.504	1.11	0.13–9.22	0.927	1.12	0.13–9.50	0.917
	T3	2.28	0.32–16.49	0.414	1.16	0.16–8.61	0.886	2.79	0.39–20.16	0.309	1.95	0.26–14.50	0.514
	T4	8.10	1.08–60.79	**0.042**	3.25	0.42–25.42	0.262	7.68	1.01–58.58	**0.049**	4.77	0.60–37.82	0.139
pN	N0	1			1			1			1		
	N1	1.72	0.98–2.99	0.056	1.25	0.68–2.29	0.467	1.41	0.83–2.40	0.206	0.78	0.44–1.40	0.406
	N2	4.38	2.65–7.21	**0.000**	2.46	1.36–4.46	**0.003**	4.22	2.55–6.96	**0.000**	2.40	1.36–4.24	**0.002**
pM	M0	1			1			1			1		
	M1	4.08	2.55–6.53	**0.000**	2.25	1.29–3.91	**0.004**	4.69	2.85–7.70	**0.000**	3.66	2.06–6.53	**0.000**
INHBA	Low	1			1			1			1		
	High	1.65	1.07–2.55	**0.024**	1.44	0.93–2.25	0.100	1.94	1.27–2.97	**0.002**	1.71	1.10–2.65	**0.017**

### Development of the nomogram

**Fig. 4:**
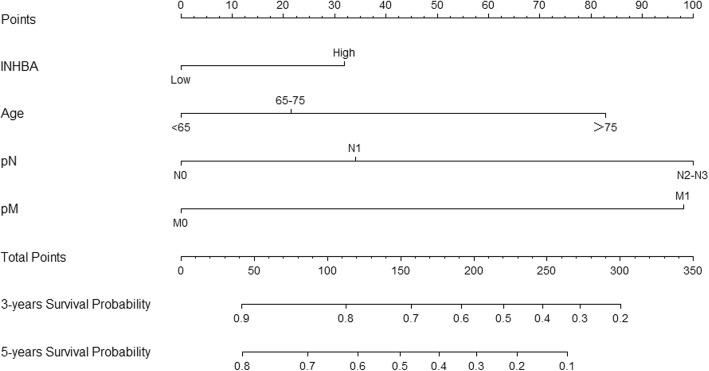
Nomogram to predict 3-year and 5-year overall survival. Each risk factor corresponded to a point by drawing a line straight upward to the points axis. The sum of the points located on the total points axis represented the probability of 3-year and 5-year overall survival by drawing a line straight down to the survival axis

**Fig. 5:**
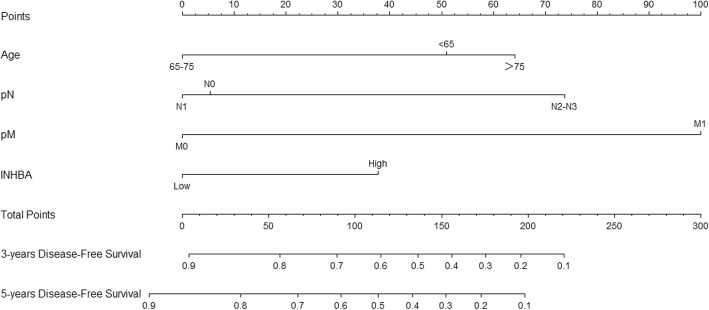
Nomogram to predict 3-year and 5-year disease free survival. Each risk factor corresponded to a point by drawing a line straight upward to the points axis. The sum of the points located on the total points axis represented the probability of 3-year and 5-year disease free survival by drawing a line straight down to the survival axis

**Fig. 6:**
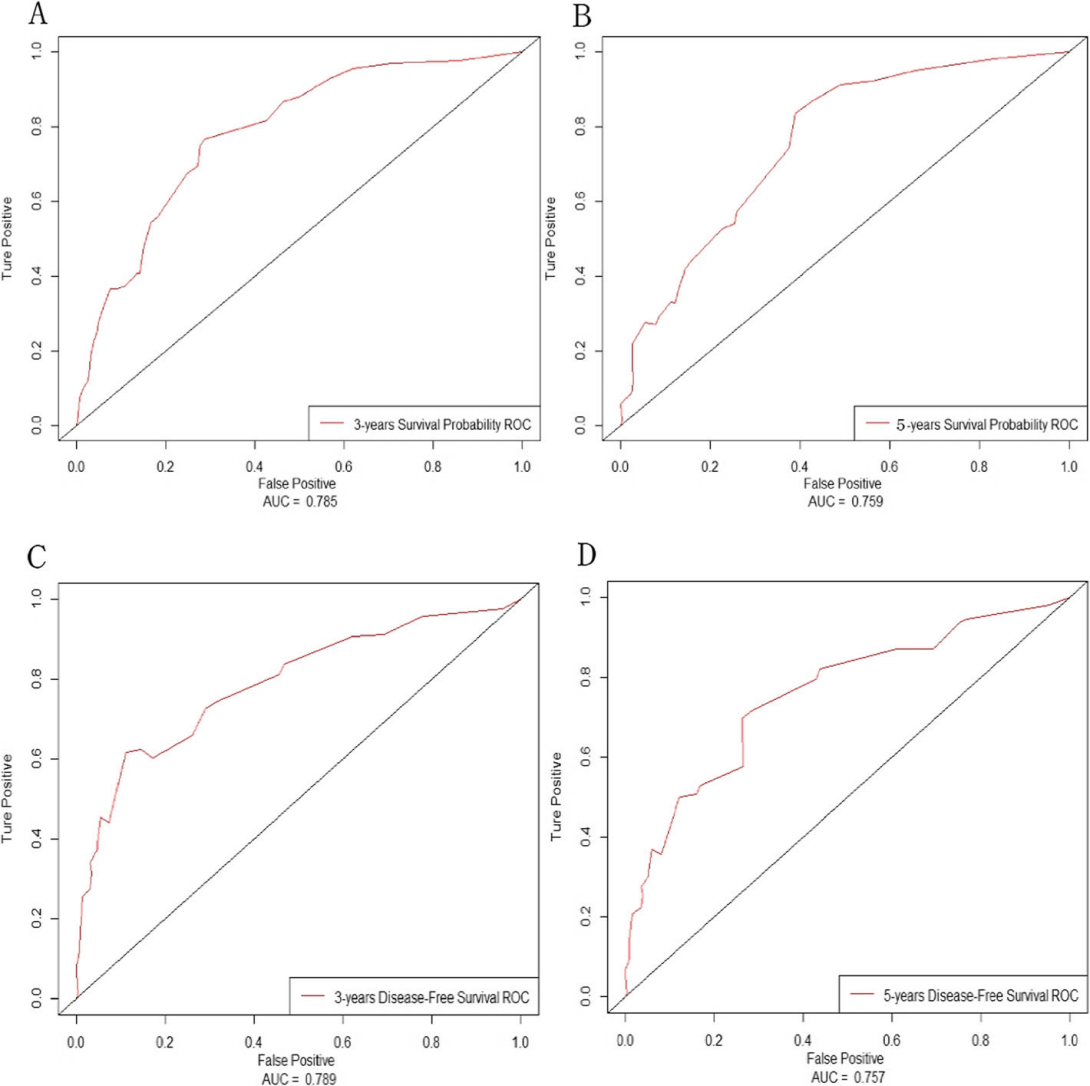
The ROC curves represented the discrimination of the models measured by the C-index: **a**. for 3-year overall survival; **b**. for 5-year overall survival; **c**. for 3-year disease free survival; **d**. for 5-year disease free survival

Based on these results, we developed two prediction models and generated graphical nomograms predicting the probability of 3-year survival and 5-year survival for OS and DFS, respectively (Figs. 4 and 5). INHBA was also included in the nomogram of OS according to the exclusion criterion of 0.1. The predictive accuracy of the nomograms calculated by AUC were 0.785 for 3-year overall survival, 0.759 for 5-year overall survival, 0.789 for 3-year disease-free survival and 0.757 for 5-year disease-free survival (Fig. 6). The calibration plots revealed good agreement between the observed outcome and predicted probability (Supplementary Figure 3).

**Fig. 7:**
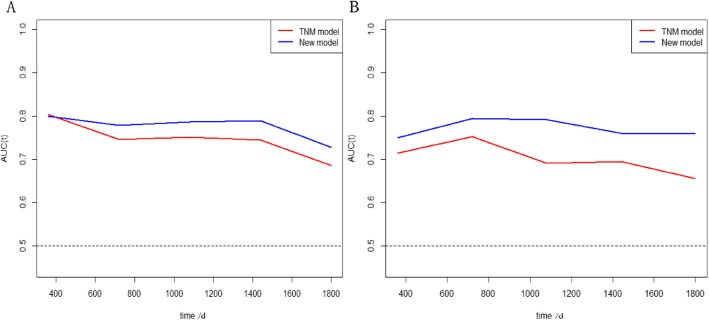
The time dependent ROC curves comparing the new models with the TNM models: **a**. Overall survival; **b**. Disease free survival

**Fig. 8:**
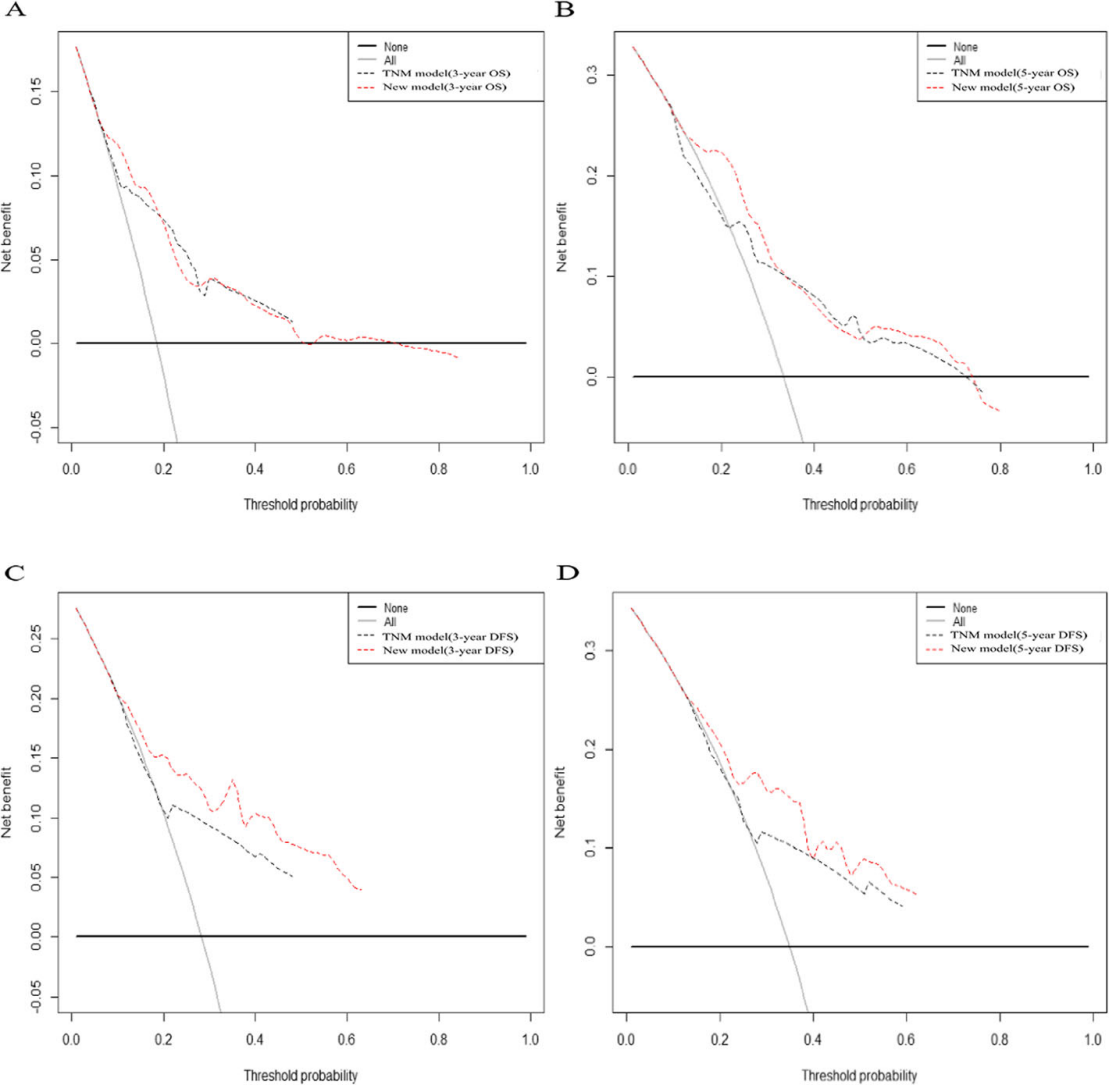
The DCA curves represented the clinical value of the models when comparing the new models with the TNM models: **a**. 3-year overall survival; **b**. 5-year overall survival; **c**. 3-year disease - free survival; **d**. 5-year disease - free survival

We also developed two prediction models based on TNM status (Supplementary Figure 4 and Supplementary Figure 5). The C-indexes were 0.748 for 3-year overall survival, 0.695 for 5-year overall survival, 0.688 for 3-year disease-free survival and 0.664 for 5-year disease-free survival. The time-dependent ROC curves also demonstrated that the predictive accuracy of new models were significantly higher than those of the TNM models (Fig. 7). The clinical value of new models were also higher than those of the TNM models for predicting disease-free survival, but were similar with that of TNM models for predicting overall survival (Fig. 8). The new models had a higher net benefit for predicting DFS than the TNM models for almost all threshold probabilities.

## Discussion

Colon adenocarcinoma is a heterogeneous malignancy with high recurrence probability and dismal prognosis. Currently, effective therapeutic strategies against recurrence and metastatic COAD remain rare. Therefore, it is important to develop a new prognostic tool to identify patients at high risk of recurrence who require more attention and treatment. It is also imperative to seek a promising therapeutic target for anti-tumor drug development to improve survival of advanced COAD.

In this study, we discovered 12 DEGs that were overexpressed in four mRNA arrays downloaded from the GEO dataset and we verified them in TCGA. According to survival analysis, INHBA was associated with both overall survival and disease-free survival. Univariate and multivariate Cox analysis were performed to process COAD clinicopathological information downloaded from TCGA. Age, pathological N and pathological M were significant independent factors for OS and DFS, while INHBA was an independent risk factor for DFS. Moreover, new graphical nomograms combining INHBA expression with clinicopathological data were generated to predict OS and DFS. For example, a 60-year-old patient (51 points) with AJCC stage TxN2 (74 points) M0 (0 points) and INHBA high expression (38 points) had a total score of 163 points. The predicted 3-year disease free survival would be approximately 37% and the predicted 5-year disease free survival would be approximately 26%. These estimates could be used in patient counseling and decision making. Finally, we developed two prediction models based on TNM status to compared with the new models. The C-index and the time-dependent ROC curves all revealed the new models had better discrimination than the TNM models. The DCA graph also indicated higher clinical value of the new model for predicting disease-free survival than the TNM model.

The mechanism of INHBA gene expression affecting outcomes in COAD remains to be fully investigated. Inhibin βA (INHBA), is a member of the transforming growth factor-β (TGF-β) superfamily, that been found to participate in invasion and metastasis in various malignant tumors [6, 19–23]. Chen et al. study found that INHBA promotes gastric cancer migration and invasion via the TGF-βsignaling pathway [21]. This pathway is involved in many processes of organismal and embryonic development, including cell growth, differentiation, apoptosis, homeostasis and others. In addition to activating the SMAD pathway, the type II receptor of TGF-βactivates the non-classical signaling pathway PI3K/AKT, thereby promoting invasion and metastasis [24]. Previous studies [25, 26] have found that oncogenes promote the Warburg effect [27] of cancer cells via the PI3K/AKT signaling pathway and affect glycometabolic reprogamming.

The TNM staging system is the foundation of prognosis prediction in colorectal cancer. Nevertheless, the prognostic power of TNM stage could be enhanced by a number of clinical, genetic and patient characteristics [28–30]. There have been several nomograms developed that predict survival for colorectal cancer. The first clinical nomogram was reported by Massacesi.et al. [31]. They used CEA, number of sites, performance status (PS) and response to first-line chemotherapy to develop a nomogram for predicting long-term survival beyond 2 years in advanced colorectal cancer. Some nomograms studies focused on CRC patients with liver metastases. Tez, et al. reported an initial US nomogram in 2008 for predicting 96-month disease-specific survival for patients with stage IV CRC after liver resection [32]. That nomogram included ten risk factors and achieved a C-index of 0.61. Kanemitsu, et al. developed a similar prognostic model for predicting death after liver resection in individuals with hepatic metastases with a C-index of 0.66 [33]. Takakura, et al. study externally validated these two models using clinical data from Hiroshima University Hospital between 1995 and 2006, and found high predictive accuracy for both nomograms [34]. Reddy, et al. also used a prognostic nomogram to evaluate peri-operative chemotherapy after resection of colorectal liver metastases [35]. Fendler, et al. focused on patients after selective internal radiation therapy of hepatic metastases [36]. Elias, et al. regarded tumor load (number of liver metastasis and peritoneal carcinomatosis index) and procedure (liver resection or/and hyperthermic intraperitoneal chemotherapy) as variables to generate a nomogram to estimate patient survival before undergoing optimal surgery [37]. This nomogram must be validated in other tertiary centers. Valentini, et al. generated brilliant nomograms based on five European randomized clinical trials for local recurrence, distant metastases and OS for patients with locally advanced rectal cancer [38]. Their study had a large sample size (2795 cases) with external validation, and the results were accurate and reliable. There are other models [39, 40] for predicting survival and recurrence in patients with rectal cancer. Nevertheless, there are no such prediction models combining genetic information with clinical data.

The present study has several advantages. First, to our knowledge, this is the first nomogram combining genetic information with clinical data for predicting survival and recurrence in patients with COAD. The tool is user-friendly for counseling patients even at the bedside. Second, we performed a head-to-head comparison with a TNM nomogram based on TCGA clinical data. The results suggest that combined consideration of genetic and clinical information could better predict prognosis. Third, we adopted a high technical standard in statistical methodology. Innovative analytical techniques were employed, including time-dependent ROC and DCA. The time-dependent ROC curve is a popular method for displaying AUC over time. DCA curves are widely used to measure clinical utility of a specific model by comprehensively considering the relative value of benefits and harms associated with the prediction model in addition to sensitively and specificity. These two methods could be interpreted simply and graphically and compared to the values of the two models better, thereby improving the accuracy of the results.

The current study also has some limitations. Despite a series of bioinformatics, we found potential prognostic genes. Nevertheless, this has not been verified using laboratory experiments. The generation of prediction models depended on a retrospective data from TCGA. The types of clinical data are limited and do not include other potential risk factors such as blood test results and underlying chronic disease. Finally, this nomogram was not validated with external data as limited by conditions.

## Conclusion

The combination of the INHBA expression signature with a clinical nomogram improves the prognostic capability in colon adenocarcinoma, especially for predicting recurrence. Further prospective studies are recommended to validate the models externally. The mechanism of INHBA in COAD need to be fully investigated.

## Supplementary information

**Additional file 1: Figure S1.** Kaplan - Meier plots of other eleven genes expression for overall survival: A. CD44; B.RFC3; C. CDK1; D. NPM1; E. MAD2L1; F. MTHFD2; G. OSBPL3; H. CSE1L; I. ATAD2; J. PMAIP1; K. PPAT.

**Additional file 2: Figure S2.** Kaplan - Meier plots of other eleven genes expression for disease free survival: A. CD44; B.RFC3; C. CDK1; D. NPM1; E. MAD2L1; F. MTHFD2; G. OSBPL3; H. CSE1L; I. ATAD2; J. PMAIP1; K. PPAT.

**Additional file 3: Figure S3.** Calibration plot. Solid line represented the current nomogram; vertical bars represented 95%CIs; the crosses indicated bias-corrected estimates: A. 3-year overall survival; B. 5-year overall survival; C. 3-year disease - free survival; D. 5-year disease - free survival.

**Additional file 4: Figure S4.** The TNM nomogram to predict 3-year and 5-year overall survival. Each risk factor corresponded to a point by drawing a line straight upward to the points axis. The sum of the points located on the total points axis represented the probability of 3-year and 5-year overall survival by drawing a line straight down to the survival axis.

**Additional file 5: Figure S5.** The TNM Nomogram to predict 3-year and 5-year disease free survival. Each risk factor corresponded to a point by drawing a line straight upward to the points axis. The sum of the points located on the total points axis represented the probability of 3-year and 5-year disease - free survival by drawing a line straight down to the survival axis.

## Data Availability

The datasets used and/or analyzed during the current study are available from the corresponding author on reasonable request.

## Abbreviations

AJCC: American Joint Committee on Cancer
COAD: Colon adenocarcinoma
CRC: Colorectal cancer
CI: Confidence interval
DCA: Decision curve analyses
DEGs: Differentially expressed genes
DFS: Disease-free survival
GEO: Gene Expression Omnibus
GEPIA: Gene expression profiling and interactive analyses
INHBA: Inhibin βA
NCBI: National Center for Biotechnology Information
OS: Overall survival
ROC: Receiver operating characteristic
TCGA: The Cancer Genome Atlas
TNM: Tumor Node Metastasis
