# Supplementary figures and images for: INHBA is a prognostic predictor for patients with colon adenocarcinoma
Source: BMC Cancer. 2020 Apr 15;20:305. doi: 10.1186/s12885-020-06743-2 (PMC7161248; doi:10.1186/s12885-020-06743-2)

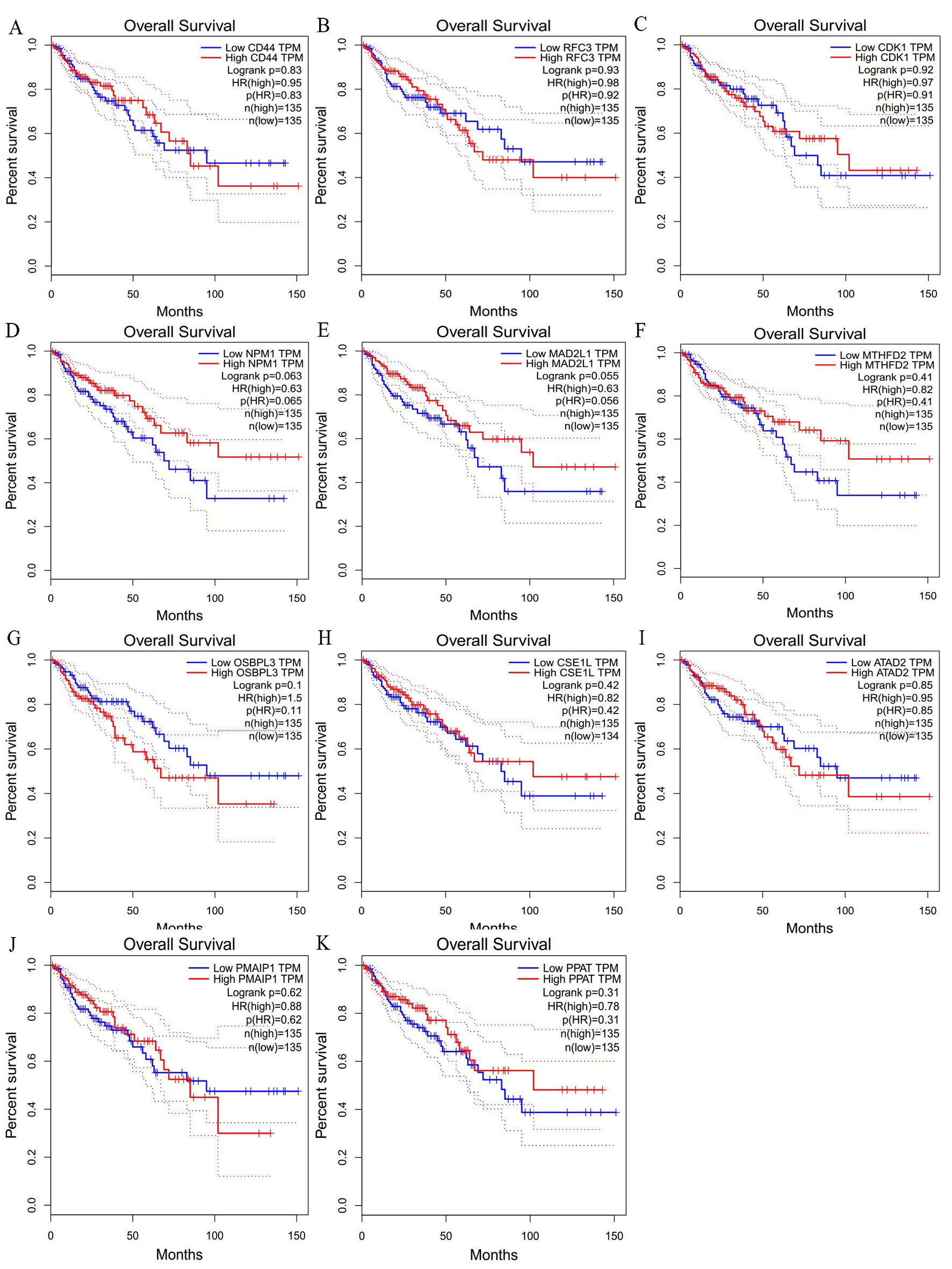

Supplement: Supplementary file 1 — Additional file 1: Figure S1. Kaplan - Meier plots of other eleven genes expression for overall survival: A. CD44; B.RFC3; C. CDK1; D. NPM1; E. MAD2L1; F. MTHFD2; G. OSBPL3; H. CSE1L; I. ATAD2; J. PMAIP1; K. PPAT. [file 12885_2020_6743_MOESM1_ESM.tif]

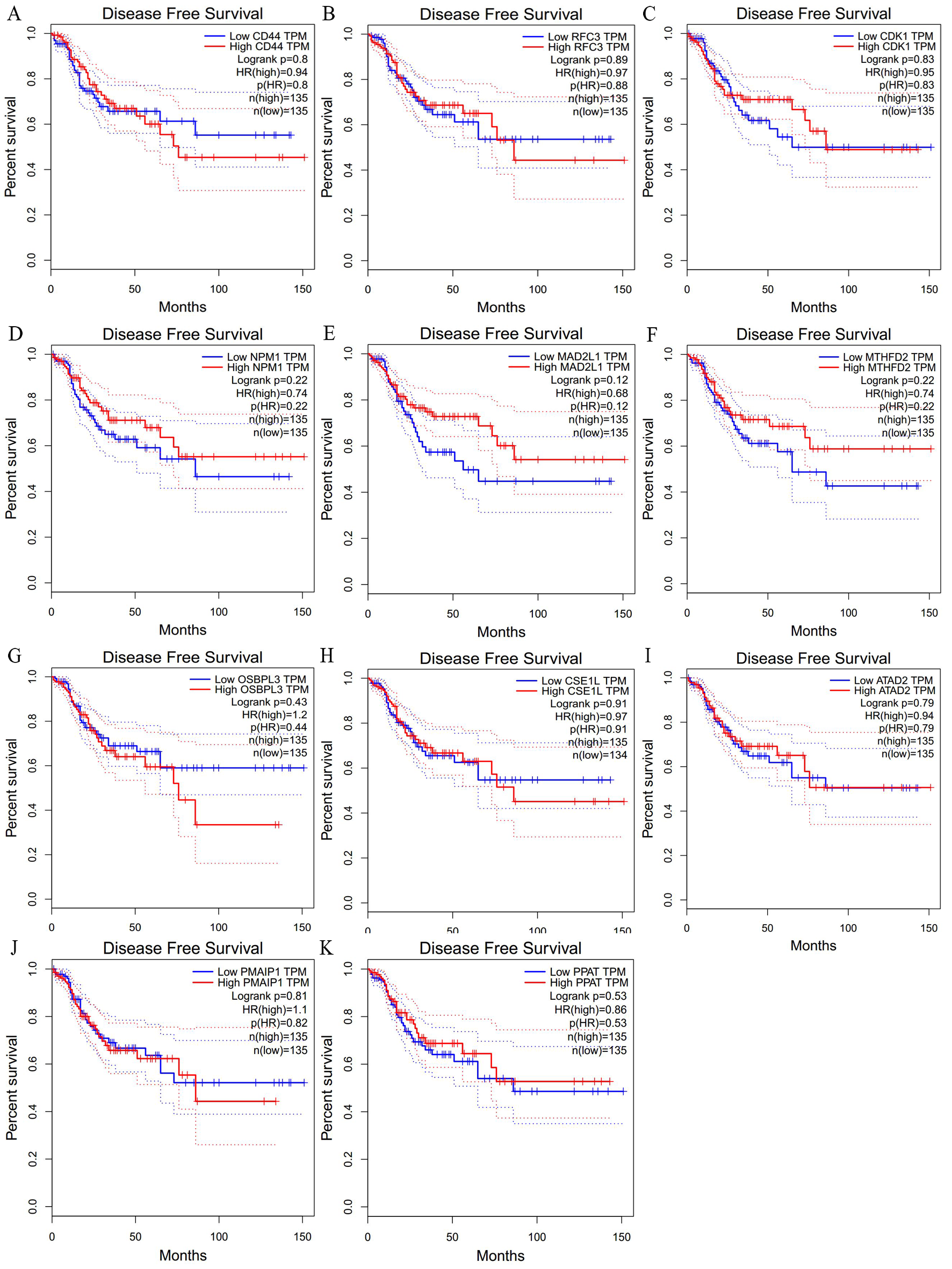

Supplement: Supplementary file 2 — Additional file 2: Figure S2. Kaplan - Meier plots of other eleven genes expression for disease free survival: A. CD44; B.RFC3; C. CDK1; D. NPM1; E. MAD2L1; F. MTHFD2; G. OSBPL3; H. CSE1L; I. ATAD2; J. PMAIP1; K. PPAT. [file 12885_2020_6743_MOESM2_ESM.tif]

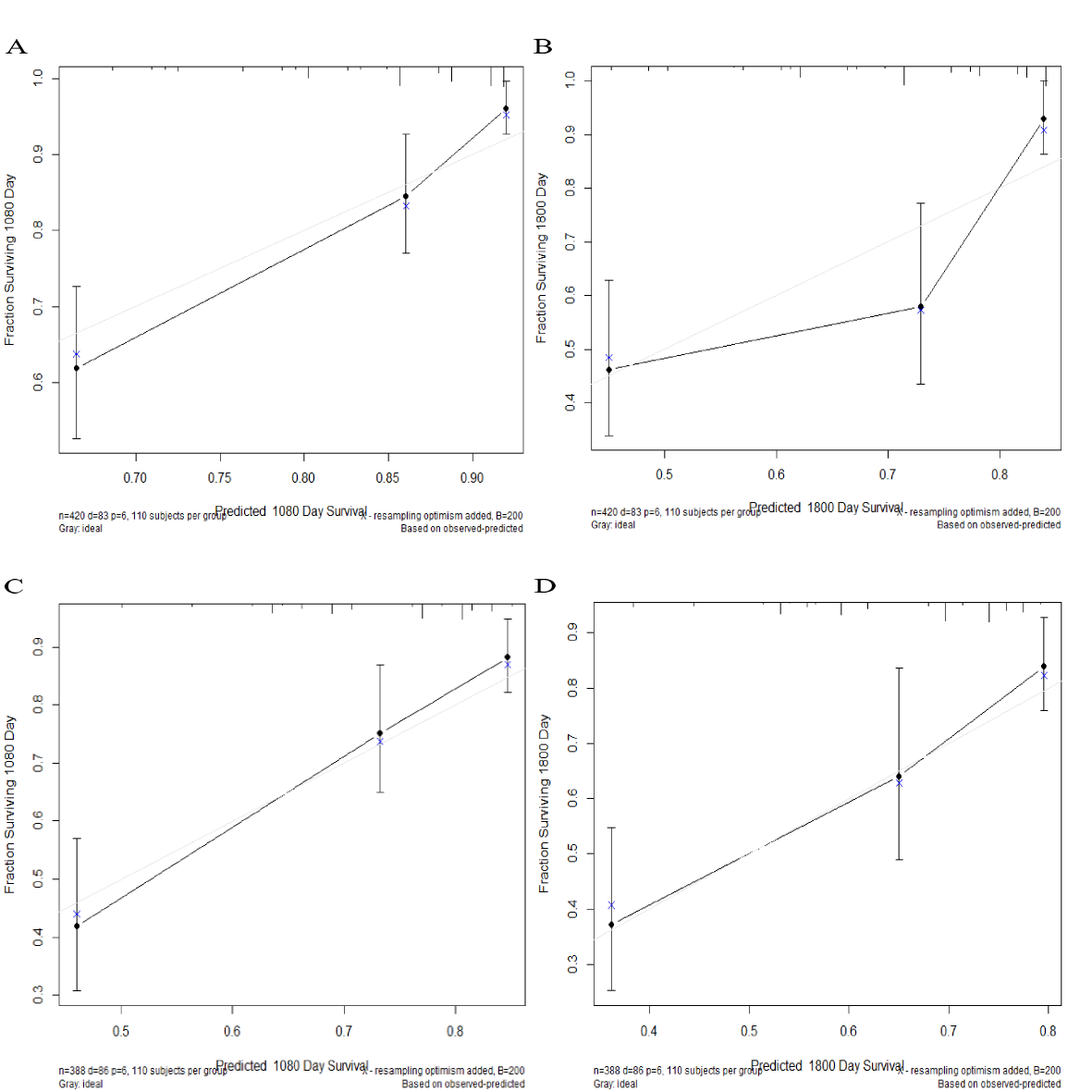

Supplement: Supplementary file 3 — Additional file 3: Figure S3. Calibration plot. Solid line represented the current nomogram; vertical bars represented 95%CIs; the crosses indicated bias-corrected estimates: A. 3-year overall survival; B. 5-year overall survival; C. 3-year disease - free survival; D. 5-year disease - free survival. [file 12885_2020_6743_MOESM3_ESM.tiff]

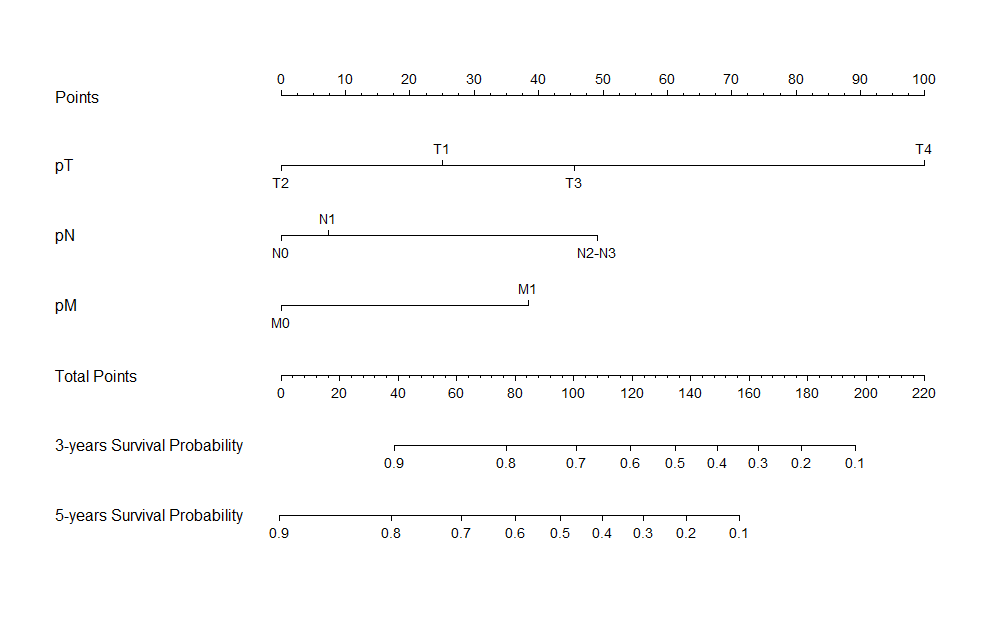

Supplement: Supplementary file 4 — Additional file 4: Figure S4. The TNM nomogram to predict 3-year and 5-year overall survival. Each risk factor corresponded to a point by drawing a line straight upward to the points axis. The sum of the points located on the total points axis represented the probability of 3-year and 5-year overall survival by drawing a line straight down to the survival axis. [file 12885_2020_6743_MOESM4_ESM.tiff]

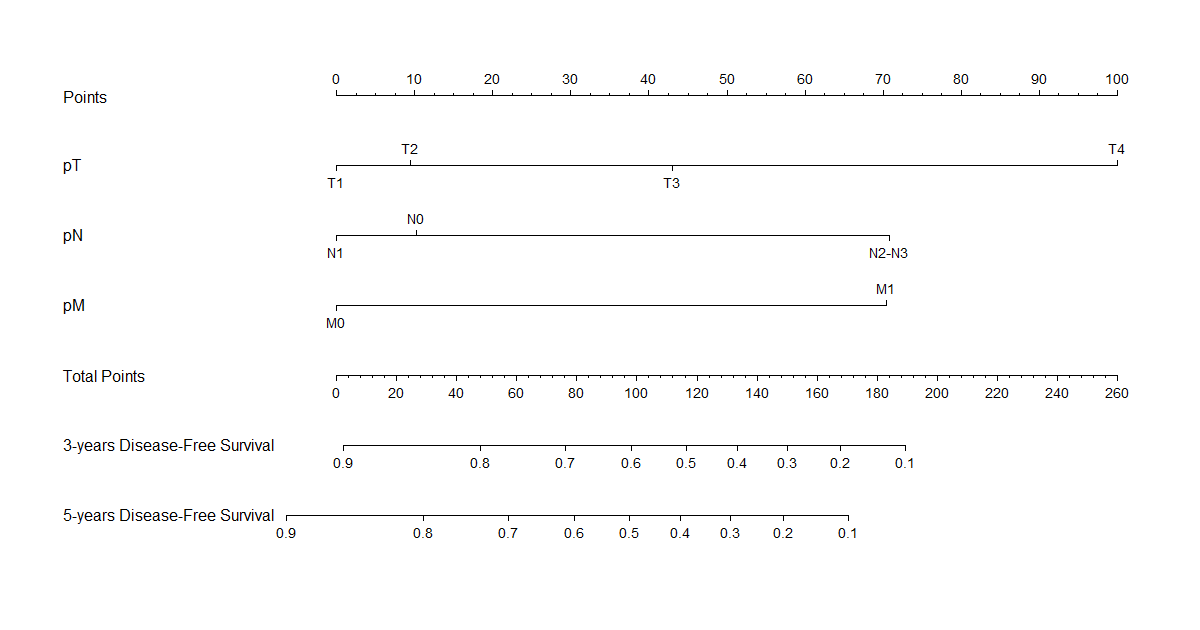

Supplement: Supplementary file 5 — Additional file 5: Figure S5. The TNM Nomogram to predict 3-year and 5-year disease free survival. Each risk factor corresponded to a point by drawing a line straight upward to the points axis. The sum of the points located on the total points axis represented the probability of 3-year and 5-year disease - free survival by drawing a line straight down to the survival axis. [file 12885_2020_6743_MOESM5_ESM.tiff]
